# Technology use and health behavior among patients with diabetes: do underlying motives for technology adoption matter?

**DOI:** 10.3389/fdgth.2025.1455261

**Published:** 2025-04-15

**Authors:** Sára Imola Csuka, Barbara Horvát, Georgina Csordás, Csilla Lakatos, Tamás Martos

**Affiliations:** ^1^ Institute of Psychology, University of Szeged, Szeged, Hungary; ^2^ Schools of PhD Studies, Semmelweis University, Budapest, Hungary; ^3^ Department of Developmental and Educational Psychology, Eszterházy Károly Catholic University, Eger, Hungary; ^4^ Faculty of Health Sciences, University of Miskolc, Miskolc, Hungary; ^5^ Faculty of Psychotherapy Science, Sigmund Freud Private University, Vienna, Paris

**Keywords:** patients with diabetes, health technology, self-determination theory, METUX model, technology adoption

## Abstract

**Introduction:**

A growing number of health technology solutions are designed for people with diabetes to ease disease self-management. However, according to some studies, technology can also bring dissatisfaction. According to the Motivation, Engagement, and Thriving in User Experience model, the use of technology is only beneficial if it is linked to the experience of autonomy. The study aimed to investigate the associations between health technology use and technology adoption motivation and associated health behavior of people with type 1 and type 2 diabetes.

**Methods:**

A cross-sectional questionnaire study was conducted on a sample of 315 patients with diabetes. The Technology Adoption Propensity Questionnaire was applied to assess general attitudes toward technology, the Autonomy and Competence in Technology Adoption Questionnaire for underlying motives of technology use, and the Summary of Diabetes Self-Care Activities tool for health behavior.

**Results:**

The results showed that technology use was predicted by proficiency (but not optimism) and lower levels of vulnerability and dependence. In addition, technology use predicted health behavior (diet and physical exercise) frequency. After refining the results further, among technology users, only autonomous motivation of technology use predicted health behavior, while controlled motivation had a slightly negative predictive effect on following the diet.

**Discussion:**

Particular attention should be paid to person-based health-related technology interventions for enhancing proficiency and reducing feelings of vulnerability and dependence on technologies. Ultimately, it is not the adoption of a technology *per se*, but the autonomous motivation for adoption that is associated with more favorable health behavior.

## 1 Introduction

The number of people living with chronic diseases is increasing. According to the WHO (1), diabetes is projected to become the seventh leading cause of death by 2030. In 2014, approximately 15% of the adult population in Hungary was found to have some kind of metabolic disorder related to diabetes, and 65% were considered overweight or obese, which are the risk factors for type 2 diabetes (2). There may also be gender differences in health behavior. The results of studies showed inconsistent findings regarding diet adherence, with some studies showing no gender differences (3, 4) while other studies found that men were more successful in weight control, which also included following the diet (5). Another study also showed that men benefited more from weight loss diabetes prevention interventions in which dietary changes and increased physical activity were important components; however, women had greater autonomous motivation (6).

The healthcare sector is now rich in opportunities to provide its users with a partnership in their treatment (7–9). The number of smartphones used worldwide has almost doubled from 3.668 to 6.378 million since 2016 (10). In Europe, 79% of the population owns a smartphone (11) and, in Hungary, 89% of the population used a smartphone in 2019 (12). Mobile applications for people with diabetes are some of the most widely used medical condition support applications (13). However, there are little data on the subjective experiences of patients using health technologies and their relationship with health behavior.

According to some studies, technology-supported disease management can be beneficial (14, 15), while other studies argued that the same technology undermines personal autonomy (e.g., giving commands or using external reinforcement strategies) (16, 17). For proper technology adoption support, it is important to investigate general technological attitudes and underlying motives of technology adaptation (18, 19).

### 1.1 New technologies in diabetes self-management

An important element of self-management for patients with diabetes is the integration of health behaviors (specifically diet and exercise) into their daily lives because proper disease management, with the active involvement of the patient, is one of the most important elements for preventing complications (20, 21). To achieve a physically and mentally favorable state, diabetes self-management education and support, medical nutrition therapy, routine physical activity, smoking cessation (when needed), and psychosocial care are recommended (22). Previous studies on technology use show that disease management mainly focused on mobile phone applications, which were found to be effective in facilitating lifestyle changes, particularly in people with type 2 diabetes (23, 24).

Despite the rapid emergence of new technologies, consumers' receptiveness varies. The Technology Adoption Propensity (TAP) construct was created by Ratchford and Barnhart (19) to capture attitudes toward technology in general. The model suggests that technological optimism and proficiency can facilitate the use of technology, whereas vulnerability and technological dependence can inhibit use. It allows the testing of a wide range of technologies, therefore, we have applied it to digital health technologies used by people with diabetes, which to our knowledge has not been investigated before.

### 1.2 Technology use and self-determination

The self-determination theory [SDT (25)] is a motivational theory that defines autonomy as the freedom of choice and the experience of self-endorsed activities (26). The opposite of autonomy is external control, when the activity is performed for extrinsic social (e.g., following medical instructions to meet the doctors’ expectations without personal involvement) or material (e.g., to look good) reasons. The Motivation, Engagement, and Thriving in User Experience (METUX) model defined by Peters et al. (18) is based on the self-determination theory and focuses on the wellbeing-enhancing element of technology, going beyond the experience of purely positive emotions (27). Up to now, few studies have examined the underlying motives (autonomous or controlled) of health technology adoption (28). According to a qualitative study among people living with diabetes, patients actively use strategies in their everyday lives to maintain autonomy and experience self-directedness in the course of disease management (29). Another study showed that the same digital health technology can be autonomy-supportive for some, while it can frustrate the autonomy of others (30). Similarly, Owens and Cribb (17) concluded that wearable devices can support personal autonomy in achieving better health, while at the same time, these can amplify health anxiety. In the case of diabetes applications, satisfaction declines in inverse proportion to the number of available features (13) and diabetes support technologies sometimes lack professional design (31). Consequently, it is crucial to take subjective experiences and the motivational aspects of technology use into account.

### 1.3 Overview of the current study

The concept of autonomously motivated technology usage is a crucial part of maintaining global health (30, 32). This study aimed to investigate the predictors of current health technology use (smartphone applications or wearable and portable devices), technology adoption motivation, and associated health behavior of individuals diagnosed with type 1 and type 2 diabetes in Hungary. Among the health behaviors, the central focus of our study was a healthy diet in general (i.e., following a healthy eating plan according to the person's perception) and physical activity. Both healthy diet and physical activity are self-management activities considered beneficial in all disease conditions (33), including type 1 and type 2 diabetes (34). Otherwise, the focus of the research was not on diabetes-related technologies and these are non-pharmacological treatment options (33). It is important to note that we focused on the patients’ subjective experiences in general, including their technology-related attitudes and motivations for technology use. Therefore, we worked with broad device categories and did not differentiate between specific types and characteristics.

First, we aimed to identify the sociodemographic (e.g. gender, age, and level of education) and disease-specific (e.g., diabetes type and duration) characteristics (hereafter referred to as personal characteristics) that predict technology usage. We then examined whether the usage of health technologies predicted health behavior. Furthermore, to gain a deeper understanding of the background factors of technology adoption, we applied the METUX model (18) to examine the motivation for adopting (autonomous vs. controlled adoption) a technology.

The study had three main questions:

(1) Which personal characteristics predict more frequent health technology usage by patients with diabetes when taking TAP and personal characteristics into account?
(2) Does health technology use predict the health behavior of patients with diabetes (weekly frequency of diet and exercise) when controlled for personal variables?
(3) Does the quality of motivation for technology use predict more adaptive health behavior of patients with diabetes (weekly frequency of diet and exercise) among users of health technology?

The following hypotheses were posed based on the theory and literature presented. Weekly frequencies of diet and exercise refer to the number of days in the previous week that the person did physical activity and followed their diet.

 *H1*: A higher level of technological optimism and proficiency is associated with more frequent technology use after controlling for the effect of personal (i.e., sociodemographic and disease-specific) characteristics.
 *H2*: A higher level of technological dependence and vulnerability is associated with less frequent technology use after controlling for the effect of personal (i.e., sociodemographic and disease-specific) characteristics.
 *H3*: The use of health technology is associated with a higher weekly frequency of diet and exercise after controlling for personal (i.e., sociodemographic and disease-specific) characteristics.
 *H4*: Autonomously motivated technology adoption is associated with a higher weekly frequency of diet and exercise after controlling for personal (i.e., sociodemographic and disease-specific) characteristics.
 *H5*: Externally motivated technology adoption is associated with a lower weekly frequency of diet and exercise after controlling for personal (i.e., sociodemographic and disease-specific) characteristics.

## 2 Materials and methods

### 2.1 Participants and procedure

The study was part of a joint research project between Semmelweis University and the University of Szeged. Participants were recruited via the mailing lists of diabetes organizations and online diabetes groups. The inclusion criteria for the study were having type 1 or type 2 diabetes, not being under psychiatric treatment, and being over 18 years of age. The study was conducted using an online data collection platform (LimeSurvey) and the research was conducted in compliance with ethical rules and authorized by the Scientific and Research Ethics Committee of the Medical Research Council (authorization number: IV/2517-2020/EKU). Before participation, subjects were provided with detailed information about the study and voluntarily agreed to participate. They were able to respond anonymously and could withdraw from the study at any time.

### 2.2 Measurements

Beyond questions on general demographic and disease-specific data (e.g., diabetes type, duration), for health technology use, we applied the following measures.

Technology adoption: To categorize technology users and non-users and understand the types of health technologies used by participants, the participants had to indicate the digital technology or application that they used *most often* to maintain their health or to prevent deterioration of their health status (predefined examples given in brackets practically defined these two broad categories). The response options were (a) smartphone applications (e.g., Google Fit, Fitbit, MyFitnessPal, and Drink Water), (b) wearable and portable devices for monitoring body functions (e.g., smartwatch, smart bracelet, and smart blood glucose meter), and (c) “other device” (with the option of adding responses in their own words). For the sake of clarity, the “other” category was not included in the analysis when a digital but not clearly online tool was mentioned; however, in most cases, the response matched one of the previous categories and it was reclassified (e.g., Samsung Health was reclassified as a mobile app). We are aware that the broad category of health technology users has a high degree of heterogeneity. Thus, we followed the general definition of wearable devices “… as digital self-tracking devices designed to improve the health, fitness and well-being of their users by collecting, analyzing and displaying biomedical data” (17).

Two self-regulation scales (i.e., autonomous and controlled adoption motivation) in the Autonomy and Competence in Technology Adoption scale [ACTA (18)] were used to measure the quality of motivation behind technology adaptation. Participants were instructed to fill out the questionnaire regarding the previously indicated health technology (1 = not at all true, 5 = very true). The autonomous motivation scale contained two subscales: intrinsic (e.g. “It is going to be fun to use.”) and identified motivation (e.g. “I believe it could improve my life.”). Controlled motivation consisted of the introjected (e.g. “It will look good to others if I use it.”) and the external regulation (e.g. “I feel pressured to use it.”) subscales. The respondents who indicated that they did not use any health-related technology skipped the ACTA and were automatically led to the next scale.

The Technology Adoption Propensity Questionnaire [TAP (19, 35)] was used to measure general attitudes toward technologies (1 = the weakest agreement, 7 = the strongest agreement). The questionnaire consisted of four scales, which were optimism (e.g., “Technology gives me more control over my daily life”), proficiency (e.g., “I enjoy figuring out how to use new technologies”), vulnerability (e.g., “I think high-tech companies convince us that we need things that we don't really need”), and dependence (e.g., “Technology controls my life more than I control technology”). According to the Hungarian validation study of the questionnaire (35), an aggregated subscale of vulnerability and dependence was applied.

The Summary of Diabetes Self-Care Activities measure [SDSCA (36)] was used to measure diabetes management activities. The questionnaire includes items about general diet (*r* = 0.55) and physical activity. The diet-related subscale consisted of two items asking whether the respondent followed a healthy eating plan in the previous week and followed their eating plan (1 = 0 days/week, 8 = 7 days/week) for an average number of days per week during the previous month. The physical activity variable used in the analyses was completed with an additional physical activity frequency variable to enhance the internal consistency of the variable [“In the last week, how many days did you do at least 30 min of physical activity? (Total duration of continuous physical activity, including walking),” Cronbach *α* = 0.77].

### 2.3 Statistical analyses

Statistical analyses were performed using the IBM SPSS Statistics version 28 software package. The statistical procedures used were preceded by a check of the conditions of applicability in all cases. After a descriptive characterization of our sample, comparisons (independent sample *t*-test for continuous variables, *χ*^2^ test for binary variables, and Kolmogorov–Smirnov *Z* test for ordinal variables) were first made at the bivariate level between the groups of non-users and users of health technology according to personal characteristics.

To test our hypotheses, multivariate procedures were used. In the first part, binary logistic regression analysis was conducted to examine predictors of actual use of health technology. In the second part, two sets of hierarchical regression analyses were conducted. In the first set, we used two regression models to examine whether technology use predicts diet adherence and physical activity. In the second set, we included only those who used health technology and examined whether the type of technology-use-motivation predicted healthy behavior.

## 3 Results

Data from 315 participants were analyzed (*n*_men_: 93, *n*_women_: 221, missing = 1). The mean age was 54.9 years (SD = 16.48, range = 19–87). Approximately one-third of the participants had a university, college, or higher degree (34.6%, *n* = 109), 13% (*n* = 41) had vocational qualifications, 30.2% (*n* = 95) had a high school diploma, and 22.2% (*n* = 70) graduated from primary school or vocational school. More than half of the respondents (53.9%, *n* = 166) reported a medium level of self-rated health, 37% (*n* = 114) rated their health status as good or very good, and only 9.1% (*n* = 28) of them rated their health status as poor or very poor (missing = 7). Of the 315 respondents, 100 reported having type 1 diabetes, and 181 reported having type 2 diabetes (missing = 35). The 297 respondents had diabetes for an average of 15.17 years (*SD* = 11.35, range = 1–58). Regarding the proportion of technology use, 56.2% (*n* = 177) of the respondents used health technology, with 53.1% (*n* = 94) of them using smartphone apps and 46.9% (*n* = 83) using wearable and portable body function monitoring devices. We followed the research questions in the analytic process and present the results in accordance.

### 3.1 Question 1

General differences were examined between non-user patients (43.8%, *n* = 138) and health technology users (56.2%, *n* = 177).

First, we compared patients with diabetes who used health technology to those who did not use any technology in terms of general sociodemographic and disease-related variables, health behavior (dieting and physical activity), and general willingness to adopt a technology. Table 1 summarizes the results of the independent-samples *t*-tests, the *χ*^2^ tests, and the Kolmogorov–Smirnov *Z* tests. The results show that, on average, technology users were younger [*t* (*df* = 306.68) = 5.29, *p* < 0.001], had higher educational attainment (Kolmogorov–Smirnov *Z* test = 1.79, *p* < 0.01), and the patients with type 1 diabetes were more likely to be technology users relative to the patients with type 2 diabetes [*χ*^2^ (*df* = 1) = 10.35, *p* < 0.01]. Regarding health behavior, technology users were more likely to follow a healthy diet [*t* (*df* = 283) = −2.04, *p* < 0.05] and exercise more frequently [*t* (*df* = 283) = −3.00, *p* < 0.01], and consequently had better overall self-rated health status (Kolmogorov–Smirnov *Z* test = 1.74, *p* < 0.01). Their attitudes were more optimistic toward technology in general [*t* (*df* = 302) = −5.78, *p* < 0.001] and rated themselves more proficient with technology [*t* (*df* = 252.73) = −7.67, *p* < 0.001].

**Table 1:** Differences between health technology users and non-users in the studied variables.

Independent sample *t*-test	Non-user		User		*t*	*df*	Effect size (Cohen's *d*)
	*m*	SD	*m*	SD			
Age	60.17	14.82	50.77	16.58	5.29**	306.68	15.83
Diabetes duration	15.69	11.53	14.75	11.22	0.69	275	11.36
Subjective financial status compared to the rest of the country (1 = bad, 10 = good)	5.33	1.81	5.89	1.94	−2.59	313	1.88
SDSCA—Diet (range: 1–8)	5.39	2.14	5.77	1.81	−1.58	248.491	1.96
SDSCA—Physical activity (range: 1–8)	3.65	1.92	4.32	1,84	−3.00**	283	1.88
TAP—optimism	2.62	1.61	3.70	1.61	−5.78***	302	1.61
TAP—proficiency	3.52	1.42	4.73	1.27	−7.67***	252.73	1.33
TAP—dependence and vulnerability	3.63	1.51	3.74	1.48	−0.614	301	1.50
*χ*^2^ test	Count	Expected count	Count	Expected count	*χ*^2^ test	*df*	
Gender							
Men	42	40.9	51	52.1	0.08	1	
Women	96	97.1	125	123.9			
Type of diabetes							
Type 1	32	44.8	68	55.2	10.35**	1	
Type 2	126	126	155	155			
Kolmogorov–Smirnov *Z* test					Kolmogorov–Smirnov *Z*		
Education level					1.79**		
Overall self-rated health					1.68**		
Types of health technologies used							

SDSCA, Summary of Diabetes Self-Care Activities; TAP, Technology Adoption Propensity.

* *p* < 0.05; **p < 0.01; ***p < 0.001.

The binary logistic regression analysis was used to examine which personal characteristics and general attitudes toward technology predicted the actual technology use (Table 2). Age [ExpB (*df* = 1) = 0.977, *p* < 0.05] and feelings of dependence and vulnerability inversely predicted health technology adoption [ExpB (*df* = 1) = 0.723, *p* < 0.01], while proficiency [ExpB (*df* = 1) = 2.161, *p* < 0.001] and having a vocational qualification [ExpB (*df* = 1) = 3.324, *p* < 0.05] positively predicted health technology adoption.

**Table 2:** Binary logistic regression of predictors of technology adoption (personal characteristics and technology adoption propensity).

Health technology use (yes/no)		*p*	OR (95% CI)
Predictors	Gender: women (men: reference category)	0.528	1.25 (0.62–2.51)
	Age	**0**.**022**	0.97 (0.95–1)
	Diabetes duration	0.337	0.99 (0.95–1.02)
	Diabetes type (type 1 diabetes: reference category)	0.458	1.37 (0.59–3.2)
	Subjective financial status	0.911	0.99 (0.84–1.17)
	Education—primary school or vocational school (reference category)	0.004	
	Education—high school diploma	0.133	0.50 (0.20–1.24)
	Education—vocational qualification	**0**.**018**	4.39 (1.29–14.94)
	Education—university or higher degree	0.501	0.74 (0.30–1.80)
	SRH—poor or very poor (reference category)	0.266	
	SRH—medium	0.728	0.80 (0.24–2.75)
	SRH—good	0.617	1.39 (0.38–5.05)
	TAP—optimism	0.155	1.19 (0.94–1.50)
	TAP—proficiency	**0**.**001**	2.20 (1.63–2.97)
	TAP—dependence and vulnerability	**0**.**024**	0.76 (0.59–0.97)

The *p*-values of significant predictors are presented in bold.

Gender: 0: female: 1: male, diabetes type: 0: type1, 1: type2, OR, odds ratio; CI, confidence interval; TAP, Technology Adoption Propensity; SRH, self-rated health.

*N* = 267.

### 3.2 Question 2

The hierarchical regression analysis was used to examine whether health technology use predicted health behavior after controlling for the effect of personal characteristics (Table 3).

**Table 3:** Hierarchical regression analysis.

Outcome variables	Model	1. Full sample (technology users and non-users) (*N* = 295)		2. Technology users (*n* = 169)	
		SDSCA_Diet (range: 1–8)	SDSCA_Physical activity (range: 1–8)	SDSCA_Diet (range: 1–8)	SDSCA_Physical activity (range: 1–8)
Predictors (standardized beta coefficients)	Block 1				
	Sex: female (male: reference category)	0.14*	0.04	0.13	0.05
	Age	0.06	0.12	−0.02	0.24*
	Diabetes duration	0.14*	−0.05	0.09	−0.03
	Diabetes type	−0.08	−0.16	−0.16	−0.18
	Block 2				
	Gender: women (men: reference category)	0.14*	0.05	0.08	0.03
	Age	0.105	0.19*	0.05	0.26**
	Diabetes duration	0.14*	−0.05	0.06	−0.05
	Diabetes type	−0.08	−0.14	−0.14	−0.15
	Technology use (non-user: reference category)	0.13*	0.24***	—	—
	ACTA_autonomous	—	—	0.32***	0.23**
	ACTA_controlled	—	—	−0.17*	−0.03
	∑ ΔR^2^ (block 2)	0.02	0.05	0.10	0.05
	*R* ^2^	0.08	0.07	0.16	0.10
	F	4.35***	3.98***	4.77***	2.6*

SDSCA, Summary of Diabetes Self-Care Activities; Autonomy and Competence in Technology Adoption.

1. Predictive effect of technology use on health behavior (controlling for the effect of sociodemographic characteristics). 2. Predictive effect of motivation to use technology on health behavior (controlling for the effect of sociodemographic characteristics).

*p < 0.05; **p < 0.01; ***p ≤ 0.001.

Both weekly healthy diet adherence (*β* = 0.13, *p* < 0.05) and exercise frequency (*β* = 0.24, *p* < 0.01) were significantly predicted by technology adoption. In addition, following a diet was predicted by gender (*β* = 0.14, *p* < 0.05, as women are more likely to follow a diet) and the duration of diagnosis (*β* = 0.14, *p* < 0.05, those living with diabetes for a longer period of time, regardless of the type of diabetes, are more likely to follow the diet). Beyond technology use, age (*β* = 0.19, *p* < 0.05, older individuals exercise more frequently) also predicted exercise frequency.

### 3.3 Question 3

Two additional hierarchical regression analyses were conducted only including individuals who used health technology (smartphone application or a wearable and portable device) (Table 3). We examined whether the quality of motivation underlying technology adoption predicts healthy behavior while controlling for other variables (personal characteristics). The results showed that following a diet was predicted by autonomous (*β* = 0.32, *p* < 0.001) and controlled (*β* = −0.17, *p* < 0.05) motivations for technology use, while personal characteristics did not have significant predictive power. The frequency of physical activity was also predicted by autonomously motivated technology adoption (*β* = 0.23, *p* < 0.01). However, controlled motivation and personal characteristics had no predictive power.

## 4 Discussion

This research aimed to investigate the complex associations between the use of health technology and health behavior in people living with type 1 and type 2 diabetes. We did not study the technology itself but rather the subjective attitudes of patients with diabetes toward these technologies and the underlying motivation for usage by applying the METUX model (18).

### 4.1 Personal characteristics and technology adoption

Our first research question was which personal characteristics predict health technology adoption of patients with diabetes. According to the results, the use of health technology was predicted by the following attitudes toward technology: high proficiency and low sense of dependence and vulnerability. In contrast, optimism which is a facilitating factor according to the TAP model (19) had no significant exploratory power. It is conceivable that merely a positive perception of technology is not enough to induce one to use such a technology. As reported by a recent study on the adoption of self-service technologies, proficiency had the strongest effect on actual use (37).

Our results confirmed that older people with diabetes are less likely to use technology to maintain their health. As previous studies showed, older age groups are less likely to adapt well to technology (38, 39). The increasing prevalence and multimorbidity of type 2 diabetes in the elderly underline the importance of supporting them in technology use (40).

The subjective financial status of respondents did not predict technology adoption. Previous studies showed that individuals with lower levels of income and with lower education are less likely to use technologies (38, 39). However, our sample collection probably did not reach the most disadvantaged groups, and considering the rise in smartphone usage in Europe in recent years (11), it appears that these technologies are less of a luxury. According to a recent study conducted in Hungary, almost 70% of homeless people owned a mobile phone and they used it for health purposes (41). Consequently, the actual use of mobile phones is less limited by lack of availability.

Actual health technology use was not predicted by diabetes characteristics (type and duration of diabetes). Consequently, diabetes type itself may not determine the adoption of health technology, which shows that these findings are widely applicable. However, the findings of recent reviews have shown that mobile phone diabetes applications are well established and applicable for type 2 diabetes, while these were not or less effective in people with type 1 diabetes (23, 24). In contrast, according to Adu et al. (42), a mobile phone intervention to support self-management was useful for both diabetes types. Further investigation is needed to determine the special requirements of each diabetes type.

### 4.2 Technology use and healthy behavior

Our second research question was about factors predicting healthy behavior (i.e., healthy diet and exercise) in people with diabetes. Health technology use was a significant predictor of the frequency of healthy behavior. Hence, those who use technology seem to be more likely to be able to use it effectively in disease management. Previous studies have suggested that health technologies can be effective facilitators of disease management (9, 23) by providing feedback and enabling self-monitoring, social networking, and regular reminders (14, 15, 43).

Some other factors besides technology use were also positively associated with healthy behavior. Those living with diabetes for longer periods were more likely to follow a diet, which suggests more established healthy behavior, and that they may be further in the process of adaptation to diabetes (44). Being a woman also predicted adherence to a healthy diet. This may be a cultural specificity as in Hungary there are higher rates of overweight men than women (40.1% of men relative to 29.2% of women) (1). This is also relevant because the rate of obesity in those with type 1 diabetes is comparable to that in the normal population (45, 46). These gender differences in health behavior require further investigation.

The frequency of physical activity was predicted by older age. It is conceivable that older people may be more likely to engage in regular physical activity, as a previous study has suggested they can devote more time to diabetes self-management and with greater regularity (4). It can be concluded that any intervention to promote health technology use should take these individual characteristics into account.

### 4.3 Technology use motivation and health behavior

We found that among individuals with diabetes who use health technology, internalization of the technology adoption predicted healthy behavior. This can reconcile contradictions between technology use and wellbeing (16, 17). While the autonomous motivation for technology use predicted both diet adherence and exercise frequency, the controlled motivation was independent of or showed negative associations with healthy behavior. From a theoretical perspective, our results provide support for the METUX model (18) among people with diabetes.

### 4.4 Limitations and future research

Our study contains some limitations. It was cross-sectional and correlational, and thus, we could not explore cause and effect relationships. For example, it is possible that a greater sense of proficiency eases the adoption of new technology, but inversely, technology adoption can also strengthen competence. A more detailed study of the antecedents of adoption processes should be conducted.

Although technology is becoming part of our everyday lives, the online data collection method may bias our findings. Nevertheless, internet users are fairly heterogeneous in terms of internet usage characteristics (47) and it is conceivable that those who use the internet are generally more competent and open to technology.

A further limitation is that we asked about health technologies (smartphone applications or wearable and portable devices) in general. Although the focus of our study was general healthy behavior and technological attitudes, this may blur potential differences between specific experiences with different technologies. However, health technology use was not predicted by the type of diabetes in previous studies (23, 24), while those with different diabetes subtypes can benefit differently from mobile phone apps promoting healthy lifestyles. It would be reasonable to investigate technologies used specifically for diabetes self-management and examine whether there are some special differences in health technology use between diabetes types. In addition, beyond diet and exercise, which are more preventive in nature, some other disease management activities (e.g., blood glucose self-monitoring) should be considered.

The motivation to use technology was examined using the METUX model (18); however, the adoption of technology is only one sphere of experience with technology. It would be worthwhile broadening the focus and examining user experiences according to what extent the technology interface or the behavior is autonomy-supportive (16, 30). In addition, targeted testing of specific health technologies through randomized control trials (48) will be needed in the future.

## 5 Conclusions

The results indicate that if the motivation to adopt a health technology is only externally motivating, it may not have positive consequences on health (30). Depending on patients' motivation, technology can deepen their dependence on external instructions, but can also serve as a way of experiencing autonomy. Motivational design should be applied to create technology interfaces that enhance autonomy and competence, fostering a sense of control and confidence in users. Particular attention should be paid to person-based health-related technology interventions for enhancing proficiency and reducing feelings of vulnerability and dependence (15, 28). It is suggested that practitioners should not only demonstrate the technology itself but also support the user's autonomy in using it when introducing a new health technology.

## Data Availability

The raw data supporting the conclusions of this article will be made available by the authors, without undue reservation.
